# Evaluation of Neuroprtective Effects of L-Carnitine and Fat Emulsion in the CVA Patients: A Prospective, Randomized, Double Blind, Clinical Trial

**DOI:** 10.22037/ijpr.2020.1100952

**Published:** 2020

**Authors:** Kaveh Kazemian, Shahram Ala, Mojtaba Mojtahedzadeh, Mahmoud Abedini, Abbas Alipour, Saeid Abediankenari, Mohammadreza Rafati, Ali Abaskhanidavanloo, Fatemeh Mohajerani

**Affiliations:** a *Department of Clinical Pharmacy, Pharmaceutical Research Center, Faculty of Pharmacy, Mazandaran University of Medical Sciences, Sari, Iran. *; b *Department of Clinical Pharmacy, Faculty of Pharmacy, Tehran University of Medical Sciences, Tehran, Iran. *; c *Department of Neurology, Bu Ali Sina General Hospital, Mazandaran University of Medical Sciences, Sari, Iran. *; d *Thalassemia Research Center, Mazandaran University of Medical Sciences, Sari, Iran.*; e *Immunogenetics Research Center, Mazandaran University of Medical Sciences, Sari, Iran*; f *Department of Anesthesiology, Bu Ali Sina General Hospital, Mazandaran University of Medical Sciences, Sari, Iran*

**Keywords:** L-carnitine, Fat emulsion, CVA, Neuroprotection, Brain biomarker

## Abstract

Cerebral infarction presents with neurological deficits caused by the death of neurons in a focal area of the brain. S100B is a biomarker that increases in brain damage. Neuroprotectives can reduce the brain sequels after neurological insult. The purpose of this study was to evaluate the neuroprotective effects of L-carnitine and Fat emulsion (Lipofundin^®^) alone and in combination in patients with ischemic stroke.

In a prospective, RCT, and double-blind study 100 patients with MCA ischemic cerebrovascular accident who were admitted in the first 24 h of injury entered the study. The patients were randomly assigned into four groups of L-carnitine, fat emulsion, L-carnitine plus fat emulsion and control. Fat emulsion 10%, 500 mL, was infused over 6 to 12 h and 1 gr of L-carnitine (10 mL of solution) was administered orally to patients in addition to common therapies, according to the American Heart Association and American Stroke Association (AHA/ASA) guidelines. The patients in the control group received only the usual treatment according to stroke guidelines. Blood samples before the intervention, then after 24 h, 48 h, and 7 days later were taken and immunoenzymatic colorimetric method was used for quantitative determination of S100B concentration in the patients’ serum.

In the within group analysis, all of our treatment interventions (except control group) have decreased S100B levels statistically significant (*P* < 0.05). Moreover, changes in observed levels of S100B before and after intervention were different between the groups and the observed differences were statistically significant (*P* = 0.01). In the GEE model, it was found that S100B levels in the L-carnitine plus fat emulsion group decreased more than the control group and this decline has been statistically significant [*P* = 0.02, 20.47 (CI 95%: 6.25-34.41)], but in comparison of L-carnitine and fat emulsion group with control group, did not reached statistical significance (*P* > 0.05).

Based on the results obtained from this study, it seems that L-carnitine with fat emulsion could lead to neuroprotective effects with a significant reduction in the S100B biomarker.

## Introduction

Cerebrovascular accidents are a general definition that refers to blood vessel problems in the central nervous system. These problems are due to a lack of proper blood supply to the brain (cerebral ischemia). Brain infarction, the stroke, includes neurological disorders that are caused by the death of neurons in different regions of the brain. In most ischemic stroke, there is a Middle Cerebral Artery (MCA) involvement. The general principles of therapy are based on the rapid diagnosis of stroke symptoms and the rapid onset of therapies and play an important role in controlling ischemic damage. The death of neurons following ischemia closely correlates with the essential role of mitochondrial metabolism. Brain ischemia leads to mitochondrial dysfunction in the absence of oxygen. These occurrences show the necessity of using neuroprotective compounds as prophylaxis (1).

Neuroprotection in general means a relative protection of neuronal structure or function (2). The goal of neural protection is the prevention of secondary complications or loss of neurons (3). Nino Stocchetti *et al* have shown neuroprotective strategies that limit secondary tissue loss and/or improve functional outcomes , identified in multiple animal models of ischemic, hemorrhagic, traumatic and nontraumatic cerebral lesions, such as reperfusion by intravenous thrombolytic agents, intra-arterial thrombolysis, mechanical thromboembolectomy, erythropoietin for anemia treatment, preserving brain perfusion in sepsis, controlled lowering of core body temperature to mitigate secondary injuries, neurorepair strategies, infusion of mesenchymal stromal cells (MSCs), volatile anesthetic agents, metabolic therapy, early parenteral administration of sex hormones because of their anti-apoptotic, anti-inflammatory, and anti-oxidant properties.

They demonstrated that despite all the disappointments, there are many new therapeutic possibilities still to be explored and tested. The outcome benefits of specific agents and interventions have not been demonstrated (4). Recently, several neuroprotective agents such as anti-CD49d antibodies have shown neuroprotective effects on acute ischemic stroke (5).

 No compelling efficacy data have been published regarding any pharmacologic or other therapies. Nonetheless the search for effective neuroprotection continues at stroke centers throughout the world (6). Increasing oxidative stress and increasing excitotoxicity are the dominant mechanisms of neuronal destruction (7). Oxidative stress leads to neuronal inflammation and is part of the therapeutic goal in all neuroprotective treatments because it causes apoptosis (2, 3, 8, 9).

Carnitine plays an important role in mitochondrial metabolism and reduces cellular damage and cell apoptosis due to the loss of oxygen in the blood (10) Fat emulsion is used as one of the major components in parenteral nutrition for the patients who are unable to have sufficient nutrition via oral diet (11). Fat emulsion as an energy source leads to increased production of heat production, reduced respiration ratio, and increased oxygen consumption. In the brain injuries (trauma or ischemia), the need for energy and metabolism of brain will increases, in this situation, fat emulsion is very energetic. But the presence of carnitine is necessary to transfer faty acids into mitochondria and generate energy in the mitochondria; otherwise, it does not appear to be beneficial for fat emulsion (16, 17, 18).

Due to the lack of similar studies on the efficacy of L-carnitine and fat emulsion in the clinical phase, the efficacy of L-carnitine and fat emulsion in this study is evaluated by effective marker for neuroprotection. 

Biomarkers such as S100B are molecules released by a variety of cells in the human body in response to damage or significant change in function. Neurologic biomarkers can show response after a specific therapy, given to ameliorate a neurologic insult (12). The S100B protein is a homodimeric protein that belongs to the family of Ca^2+^ - Zn^2+^ mediated proteins (13). The S100B dimer was thought to be specific to the CNS, but recent evidence indicates that it is also present in the human gut, specifically in enteric glial cells. S100B has intracellular and extracellular targets and has autocrine and paracrine effects on glia, neurons, and microglia.

All physiological actions (neurotrophic effects) appear to be exerted at nanomolar concentrations; in contrast, micromolar concentrations of the protein are found after cell damage. S100 has been considered a marker of generalized blood–brain barrier (BBB) dysfunction because it is present and secreted by astrocytes into the CSF after CNS injury (14).

## Experimental

*Study design and setting*

A prospective, RCT, and double-blind study was conducted in the intensive care unit, neurology and emergency wards of an educational hospital on MCA ischemic cerebrovascular accident (CVA) patients who were admitted in the first 24 h of injury.

The patients who met the inclusion criteria were randomly assigned, using a simple randomization procedure, for 1 to 4 treatment groups (L-carnitine, fat emulsion, L-carnitine plus fat emulsion and control). The allocation sequence was concealed from the researcher enrolling and determining allocation by using sequentially numbered opaque envelopes. The neurologists, healthcare providers, and data collectors were aware of the patients’ allocations, but the outcome assessors and data analysts remained blinded to this.

All patients received standard treatment according to the American Heart Association and American Stroke Association (AHA/ASA) guidelines such as ASA and Heparin. Fat emulsion 10% (Lipofundin® MCT/LCT, BǀBraun, Germany), 500 mL, was infused over 6 to 12 h and 1 gr of L-carnitine (10 mL of solution) (L-carnite®, Alborz daru, Iran) was administered orally to the patients (15,16). Therapeutic interventions continued for one week. Five milliliter of the patients’ blood as a base sample were taken after confirming the diagnosis of ischemic CVA by a neurologist, also sampling from the patients’ blood continued at intervals of 24 h, 48 h and, the 7th day after starting intervention, to evaluate the S100B biomarker. Serum blood samples were stored after centrifugation in a refrigerator at -70 °C in hospital laboratory. According to Diamtera’s protocol (www.diametra.com) the immunoenzymatic colorimetric method was used for quantitative determination of S100B concentration in human serum and collaborator who evaluating laboratory results did not aware the type of therapeutic intervention groups. 

*Patients*

**Figure 1:**
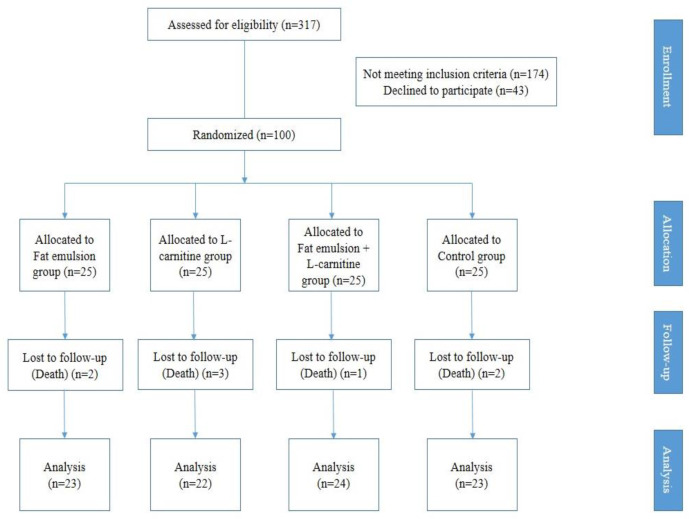
CONSORT flow diagram of the study design for enrollment, allocation, follow-up and analysis

A total of 317 patients with diagnosis of ischemic CVA were introduced by the neurology service to examine the inclusion criteria. Of these, 174 patients were excluded from the study, and 43 patients did not consent to the study, and 100 patients were evaluated (Figure 1). None of the patients didn’t exclude because of death or any ADR during this study.

The study was approved by the research ethics committee of Mazandaran University of Medical

Sciences (IR.Mazums.Rec.95-1982) and registered in the Iranian Registry of Clinical trials

under registration number IRCT2015-10043014N11 (the full trial protocol can be accessed at:

http://www.irct.ir). The study was performed according to the Declaration of Helsinki, and written informed consent was obtained from all the patients or first-degree relatives of them before their enrollment in the study.

*Inclusion criteria*

All patients with MCA ischemic stroke that hospitalized within 24 h after their symptoms and age between 21 to 75 years old were included.

*Exclusion criteria*

- All the patients under the age of 21 and over 75 years old, liver failure (increased liver enzymes, more than 5 times of normal range or cirrhosis), valproate consumers, patients who are under hemodialysis, ARDS, sepsis, hyperlipidemia (TG > 400), acute pancreatitis with hyperlipidemia, patients with platelets less than 70,000, hospitalization more than 24 h after symptoms appear, CNS infection, patients with a history of stroke, seizure disorders and migraine, were excluded from the study

We used the Shapiro-Wilk test to determine whether data were normally distributed. Descriptive baseline characteristics for the four groups (L- carnitine, fat emulsion, L- carnitine + fat emulsion, and control) were tabulated as mean (SD), or percentages. Comparisons between the four groups for categorical data were statistically analyzed using the chi-square; the ANOVA test or Kruskal-Wallistest were used for continuous data. The primary efficacy data on S100B was examined using intention-to-treat analysis. The General Linear Model (GLM) scores of s100b between the 4 groups were compared by a repeated measurement analysis of variance (ANOVA) test. The time of evaluation was considered a within-subject factor and intervention state (L- carnitine, fat emulsion, L- carnitine plus fat emulsion, and control), a between-subject factor. The time group (interaction term) was considered as group differences (between four groups) in their response over time. Mauchly’s sphericity test was used to verify the compound symmetry assumption. Additionally, a generalized estimating equation (GEE) model was used to estimate the differences in values for the s100b at each time point between the 4 groups and also the time trend after treatment. A *P* value of 0.05 or less was considered statistically significant. The data were analyzed using IBM SPSS Version 16 and Stata version 12 (SPSS/IBM; Armonk, New York, USA). 

## Results

In this study, 100 patients were evaluated (57 male, 43 female). Their age ranged between 45-75 years and their average age (standard deviation) was 62.43 (8.32) years.

The demographic characteristics of the patients in this study are presented in Table 1. The severity of stroke at baseline was assessed using the National Institutes of Health Stroke Scale (NIHSS). 

(SD) of S100B levels before and after intervention in each of the studied groups is presented in Table 2.

**Figure 2:**
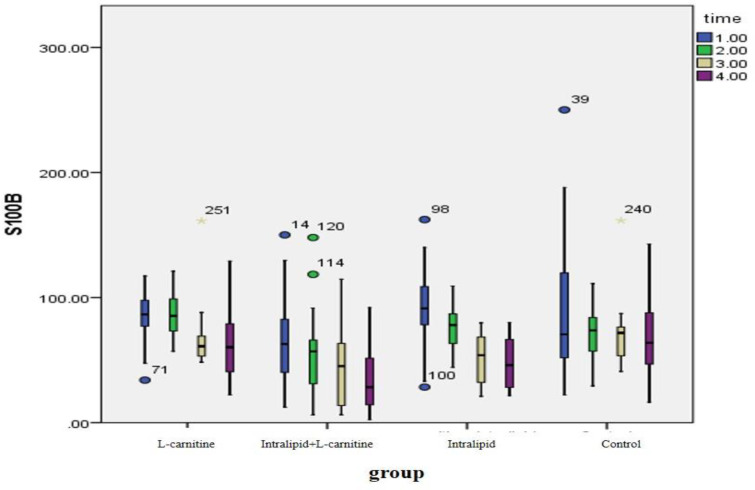
Comparison of the effects of the interventions on the reduction of S100B levels

As shown in Table 2, all our treatment interventions have decreased S100B levels and the observed differences have been statistically significant (*P* < 0.05) but in the control group, the observed changes were not statistically significant (*P* > 0.05). The changes in the observed levels of S100B before and after intervention were different between the groups and the observed differences were statistically significant (*P* = 0.01). After eliminating the effect of gender, BMI and HTN in the statistical GEE model, it was found that S100B levels in the fat emulsion plus L- carnitine group decreased more than the control group and this decline has been statistically significant [20.47 (CI 95%: 6.25-34.41)]. There was no significant difference between the groups receiving L-carnitine or fat emulsion and the control group (Figure 2).

**Table 1:** Demographic characteristic of the patient’s study.

	**Group**				***p*** ** Value**
	**L-carnitine (n = 25)**	**Lipofundin (n = 25)**	**L-carnitine + Lipofundin (n = 25)**	**Control (n = 25)**	
Age, Mean, SD	62.48 (8.76)	63.24 (9.35)	60.56 (8.55)	63.44 (6.54)	0.61
Sex, F/M	14/11	14/11	7/18	8/17	0.08
BMI, kg/m², Mean, SD	24.81 (4.03)	23.76 (2.83)	26.48 (3.63)	25.20 (4.37)	0.09
Undergraduate education, n (%)	14 (56)	17 (68)	12 (48)	14 (56)	0.56
Married, n (%)	21 (84)	23 (92)	23 (92)	23 (92)	0.72
IHD, n (%)	5 (20)	6 (24)	2 (8)	2 (8)	0.26
DM, n (%)	1 (4)	0 (0)	3 (12)	1 (4)	0.26
HTN, n (%)	10 (40)	13 (52)	5 (20)	4 (16)	0.02
DLP, n (%)	10 (40)	10 (41.7)	6 (24)	7 (28)	0.47
NIHSS, Mean (SD)	8.48 (3.19)	8.88 (2.93)	7.44 (2.86)	8.24 (2.8)	0.38

Body Mass Index (BMI), *Ischemic Heart Disease (IHD), Diabetes Melitus (DM), Hypertension (HTN), Dislipidemia(DLP),* National Institutes of

Health Stroke Scale (NIHSS), Standard deviation (SD)

**Table 2:** Mean S100B levels before and after intervention in each groups

		**Time**				**Within group effect**	**Between group effect**
		Base	24 h	48 h	7^th^ day		
S100B, mean (SD)	L-carnitine	85.87 (26.04)	86.74 (19.03)	70.55 (32.14)	64.98 (32.71)	0.01	0.01
	Fat emulsion	86.78 (34.33)	80.03 (16.03)	51.86 (21.81)	30.71 (9.60)	0.004	
	L-carnitine + Fat emulsion	68.64 (37.15)	55.96 (36.07)	45.04 (29.01)	34.58 (26.73)	0.001	
	Control	80.74 (52.40)	68.84 (19.85)	75.54 (32.49)	65.41 (40.17)	0.22	
	*p* Value	0.01	0.001	0.03	0.004		

## Discussion

The most important therapeutic strategy in the patients with cerebrovascular accident is early detection and prevention of neuronal death. In this study, considering the mechanisms involved in the death of neurons in cerebrovascular accidents, the neuroprotective effects of L-carnitine and fat emulsion with monitoring of brain biomarker levels have been investigated. Animal studies have confirmed the efficacy of these drugs, but in the clinical phase, they have not evaluated the neuroprotective effects of these drugs (11, 23, 29, 30, 31, 32). Regarding the direct effect of oxidative stress on neuronal degeneration and positive role of L-carnitine and fat emulsion in reducing oxidative stress and oxygen supply, the neuroprotective effects, Synergistic effects, and comparison of these effects in each of the studied groups have been investigated in the present study.

Carnitine is shown to have important functions in some metabolic processes such as oxidation of long-chain fatty acids (17).

L-Carnitine primarily functions to transport activated long chain fatty acids (long chain fatty acyl-CoAs) into the mitochondria for degradation by β-oxidation (17, 18, 19). In recent years, there has been considerable interest in the therapeutic potential of L-carnitine and acetyl-L-carnitine (ALCAR) for neuroprotection (1, 17, 18, 19). In addition, in the patients with chronic stroke who have muscle wasting due to an abnormality in the mitochondrial energy metabolism of their muscles, L-carnitine reduces muscle damage during rehabilitation (33, 34).

At the time of brain injuries (trauma or ischemia), the brain’s metabolism is high and the brain’s need for energy increases. In this situation, the fatty acids are very energetic and effective. But the presence of carnitine is essential for energy production in the mitochondria and in the absence of carnitine, the beneficial effects of fatty acids do not appear (17, 18, 19).

All physiological actions (neurotrophic effects) of S100B appear to be exerted at nano molar concentrations; in contrast, micromolar concentrations of the protein are found after cell damage. S100B has been considered as a marker of generalized blood–brain barrier (BBB) dysfunction because it is present and secreted by astrocytes into the CSF after CNS injury (14).

Sustained elevations of S100B have been reported in the patients with large brain infarcts (25). S100b increases in the patients with ischemic stroke. Lamers KJ *et al.,* measured by S100b in the patients with neurological disorders, found that this biomarker increased in cerebrovascular accidents (28).

 Ahmed *et al* cited the effects of L-carnitine neuroprotection on its effect on reducing S100b levels (20). Herrmann *et al*., explained this feature of L-carnitine to inhibit neuronal degeneration by reducing production of S100b. The levels of S100b are directly related to the amount of brain damage (21). H.M. Ghanem *et al.* considered oxidative stress and free radical production as a cause of increased production of S100b from astrocytes, and showed that the anti-oxidant effect of L-carnitine is responsible for reducing blood levels of S100b (22). Gianni biolo *et al.* in their study showed that carnitine 2gr/day IV for 2 weeks increases the beta-oxidation of long chain fatty acids by stimulating their passage from the mitochondrial membrane. Due to the effect of beta-oxidation of fatty acids on neural protection, it is anticipated that L-carnitine will have effects on neuroprotection with the transfer of fatty acids into mitochondria (23). Sima and BASARSLAN confirmed neurological protection of L-carnitine in their studies (15, 24).

The blood levels of the biomarker S100B were monitored as a predictor of brain degeneration and the effect of neuroprotection of interventions.

In this study, whitin group analysis, the neuroprotection was observed with a significant reduction of s100b (*P* < 0.05). 

Zimmer and Basarsalan both showed improvement in neurologic status in separate studies of fat emulsion effectiveness (11, 26).

In this study, within group analysis, showed a significant decrease in S100B in L-carnitine plus fat emulsion group in comparison to control group, indicated that neuroprotection was statistical significant (*P* <0.05) but we didn’t observe such changes in carnitine or fat emulsion group alone. (*P *> 0.05)

In 2018, Ata Mahmoodpoor *et al*., have shown in their study that there is no differences in brain biomarker or mortality following TBI after L-carnitine use (27). In this study, for the first time, the neuroprotective effects of these drugs alone and in combination, in the human phase were investigated. Our main finding in this study was a significant reduction in blood serum levels of s100b in the patients receiving L-carnitine plus fat emulsion.

As in the results section; however, in within group analysis, all interventions resulted in a significant decrease of s100b (*P* <0.05), But, between group analysis showed a significant difference at the level of s100b between the studied groups (*P* = 0.01), The group receiving fat emulsion with L-carnitine had a greater reduction than other groups and this difference was statistically significant (*P* = 0.02) (20.47 [CI 95%: 6.25-34.41]).

## Conclusion

Regarding the mechanisms involved in neuronal damage in stroke, it seems that neural protection of L-carnitine plus fat emulsion confirmed this hypothesis that L-carnitine, reduces neuronal damage, through the transfer of fatty acids into mitochondria for degradation by β-oxidation (17, 18,19,33). 

Therefore, it seems that the combination of two drugs, L-Carnitine and fat emulsion, could significantly lead to neuroprotection in patients with ischemic stroke.
